# High-Salt Loading Downregulates Nrf2 Expression in a Sodium-Dependent Manner in Renal Collecting Duct Cells

**DOI:** 10.3389/fphys.2019.01565

**Published:** 2020-01-21

**Authors:** Mi Liu, Mokan Deng, Qimei Luo, Xianrui Dou, Zhanjun Jia

**Affiliations:** ^1^Department of Nephrology, Shunde Hospital, Southern Medical University (The First People’s Hospital of Shunde), Foshan, China; ^2^Nanjing Key Laboratory of Pediatrics, Children’s Hospital of Nanjing Medical University, Nanjing, China

**Keywords:** nuclear factor E2-related factor 2, high salt, oxidative stress, chronic kidney diseases, collecting duct cells

## Abstract

**Background:**

High salt intake is associated with both oxidative stress and chronic kidney disease (CKD) progression. Nuclear factor E2-related factor 2 (Nrf2) is a transcriptional factor regulating the antioxidant and detoxifying genes to potently antagonize oxidative stress. This study examined the effect of high salt loading on the expression of Nrf2 in kidney.

**Methods:**

Mice were treated with acute salt loading, and Nrf2 expression in the kidney was detected by Western blotting and immunostaining. Reactive oxygen species (ROS) levels in the kidney were measured using dihydroethidium (DHE) staining. *In vitro*, mpkCCD cells were cultured in high osmolality medium by adding sodium chloride (NaCl), sodium gluconate (Na-Glu), choline chloride (Choline-Cl), or mannitol. Then, Nrf2 and its target genes were measured.

**Results:**

Nrf2 protein in renal cortex and medulla tissue lysates was significantly downregulated after acute salt loading. Immunofluorescence data showed that Nrf2 was mainly located in collecting duct principal cells evidenced by co-staining of Nrf2 with AQP2. Contrasting to the reduced Nrf2 expression, ROS levels in the kidney were significantly increased after salt loading. *In vitro*, the Nrf2 protein level was downregulated in mpkCCD cells after NaCl treatment for 24 h. Interestingly, sodium gluconate had a similar effect on downregulating Nrf2 expression as NaCl, whereas neither Choline-Cl nor mannitol changed Nrf2 expression. Meanwhile, the mRNA levels of Nrf2 target genes were downregulated by NaCl and/or sodium gluconate, while some of them were also regulated by Choline-Cl, indicating a more complex regulation of these genes under a high salt condition. Finally, we found that the downregulation of Nrf2 caused by NaCl was not affected by N-acetylcysteine (NAC), spironolactone, or NS-398, suggesting other mechanisms mediating Nrf2 downregulation caused by high salt challenge.

**Conclusion:**

High salt downregulated Nrf2 mainly via a sodium-dependent manner in kidney collecting duct cells, which might contribute to the excessive renal oxidative stress and CKD progression.

## Introduction

Chronic kidney disease (CKD) is one of the most serious health problems affecting millions of people and draining health care resources. A cross-sectional survey showed that the overall prevalence of CKD in China was 10.8% (Zhang et al., 2012). Inappropriate lifestyle habits, especially dietary habits such as excessive salt intake, accelerate CKD via blood pressure (BP)-dependent and -independent mechanisms (Hosohata, 2017). A number of clinical studies demonstrated that high sodium intake was associated with CKD progression in patients in early stages of CKD (Nerbass et al., 2015; Leonberg-Yoo and Sarnak, 2017; Malta et al., 2018). Moreover, animal studies also confirmed this notion. Cao et al. (2015) reported that high salt activated the renal and cerebral renin–angiotensin axis and thus promoted the progression of CKD independently of BP in 5/6-nephrectomized rats. Likewise, a high-salt diet induced outward remodeling of efferent arterioles and led to lower glomerular filtration rate (GFR) in mice with reduced renal mass (Zhao et al., 2017). In spite of the well-recognized association between high-salt diet and CKD progression, the mechanisms remain not fully understood. A high-salt diet induces renal oxidative stress and kidney injury in Dahl rats (Huang et al., 2016). An *in vitro* study also demonstrated that high sodium chloride (NaCl) increased reactive oxygen species (ROS) in mouse renal inner medullary cells (mIMCD3) (Zhang et al., 2004). It is well known that oxidative stress is a driver of CKD progression, cardiovascular disease, and other complications (Vaziri, 2004; Ruiz et al., 2013; Tan et al., 2015). Thus, it could be speculated that high-salt-induced oxidative stress might be involved in the pathogenesis of CKD to some degree. However, the detailed mechanism mediating high-salt-induced oxidative stress is still elusive.

Nuclear factor erythroid 2-related factor 2 (Nrf2) is a redox-sensitive transcription factor that regulates cellular responses to oxidative stress. Under normal physiological conditions, Nrf2 is sequestered in the cytoplasm by Kelch-like ECH-associated protein 1 (KEAP1), which promotes the proteasomal degradation of Nrf2 (Itoh et al., 1999). When cells are exposed to stimuli such as electrophilic compounds, ROS, and endoplasmic reticulum (ER) stress, KEAP1 undergoes a conformational change. Such a change can cause the dissociation of Nrf2 from KEAP1, leading to the translocation of Nrf2 into the nucleus, where Nrf2 can bind to the antioxidant response element (ARE) and consequently activate ARE-dependent gene expression of a series of antioxidative and cytoprotective proteins including NAD(P)H:quinone oxidoreductase 1 (NQO1), heme oxygenase 1 (HO-1), aldo-keto reductases (AKR), and glutathione S-transferase (GST) (Kobayashi and Yamamoto, 2005; Hayes and Dinkova-Kostova, 2014; Penning, 2017). Because oxidative stress is a major pathogenic factor for kidney diseases, the Nrf2 system has been proposed to be a therapeutic target for renal protection. Nrf2 activators were reported to be protective against oxalate-induced nephrolithiasis (Zhu et al., 2019), endothelial dysfunction in CKD (Aminzadeh et al., 2013), and ischemia–reperfusion injury (Liu et al., 2014). However, it remains unknown if high salt could dysregulate Nrf2 system. In the present study, we aimed to investigate the effect of high salt on regulating the expression of the Nrf2 system and the underlying mechanism.

## Materials and Methods

### Animal Studies

In all studies, 3- to 4-month-old male mice with a C57BL/6J genetic background were purchased from Guangdong Medical Laboratory Animal Center. All mice were maintained under a 12:12-h light–dark cycle (lights on at 6:00 a.m. and lights off at 6:00 p.m.). Acute salt loading was performed as described previously (Jia et al., 2006). Briefly, the mice were first intraperitoneally injected with 1 ml of water and then administrated with a single dose of 1 mEq of Na^+^ in the hypertonic solution by oral gavage. The control mice were treated with the same volume of water. After 6, 12, and 24 h, mice were sacrificed, and the renal tissues were harvested for the evaluation of gene and protein expressions and histological analysis. All procedures were in accordance with the guidelines approved by the Institutional Animal Care and Use Committee at Nanjing Medical University.

### Immunohistochemistry

The kidneys were fixed with 10% formalin and embedded in paraffin. Kidney sections (4 μm in thickness) were incubated in 3% H_2_O_2_ for 15 min at room temperature to block endogenous peroxidase activity. After boiling in antigen retrieval solution (1 mmol/L Tris–HCl, 0.1 mmol/L EDTA, pH 8.0) for 15 min at high power in a microwave oven, the sections were incubated overnight at 4°C with a rabbit anti-Nrf2 antibody (Santa Cruz, Dallas, TX, United States). After washing with PBS, the secondary antibody was applied, and the signal was visualized using an ABC kit (Santa Cruz, Dallas, TX, United States).

### Immunofluorescent Staining

The kidneys were fixed with 10% formalin and embedded in paraffin. After deparaffinization, kidney sections (4 μm thickness) were processed for double labeling with immunofluorescence. The slides were blocked in 1% BSA for 1 h and then co-incubated with rabbit anti-Nrf2 and goat anti-AQP2 antibody (Santa Cruz, Dallas, TX, United States) at 4°C for overnight. After washing off the primary antibody, slides were incubated for 1 h at room temperature with donkey anti-rabbit IgG-TRITC and donkey anti-goat IgG-FITC (Santa Cruz, Dallas, TA, United States). Resulting slides were scanned and quantified using Image-Pro Plus 6.0 software. Kidneys from four to five mice per group were analyzed.

### DHE Staining

The ROS levels in the kidney were measured with the fluorescent probe dihydroethidium (DHE). Fresh frozen kidney sections (−26°C, 10 μm thickness) were incubated with 1 μM DHE (Bestbio, China) for 30–60 min at 37°C. Digital images of 10 random fields from each sample were taken using an Olympus fluorescence microscope (magnification × 400) and the fluorescence intensity was analyzed using Image-Pro Plus 6.0 software.

### Cell Culture Experiments

Immortalized mouse kidney cortical collecting duct cells (mpkCCD) were purchased from BioVector NTCC Inc. Cells were routinely propagated in DMEM-F12 supplemented with 10% fetal bovine serum and 1% Pen-Strep solution and maintained at 37°C in 5% CO_2_ in a humidified incubator as described previously (Bens et al., 1999). Cells were grown to 80–90% confluence and starved for 12 h with DMEM-F12 medium that contained no drugs or hormones before treatment. NaCl was prepared by adding 125 mM NaCl to DMEM-F12 to increase osmolality to 550 mOsm/kgH_2_O. Mannitol was prepared by adding 240 mM mannitol to DMEM-F12 to increase osmolality to 550 mOsm/kgH_2_O. Sodium gluconate (Na-Glu) (550 mOsm/kgH_2_O) and choline chloride (Choline-Cl) (550 mOsm/kgH_2_O) were prepared by adding 120 mM Na-Glu or choline-Cl, respectively, to isotonic medium. Cells were starved for 12 h and pretreated with serum-free medium containing N-acetylcysteine (NAC), spironolactone or NS-398 for 1 h. Cells were then treated with normal medium or high salt (HS) medium. At the end of the experiments, cells were harvested for the preparation of whole cell lysate. For all experiments, the cells had a passage number less than 20.

### Immunoblotting

The renal tissues were lysed using RIPA buffer containing 50 mM Tris (pH 7.4), 150 mM NaCl, 1% NP-40, 0.5% sodium deoxycholate, 0.1% SDS, and proteinase inhibitor. The protein concentration was determined with a Pierce BCA Protein Assay Kit (Thermo Fisher Scientific). Protein (60 μg) from renal lysates was denatured in boiling water for 10 min, separated via SDS-polyacrylamide gel electrophoresis, and transferred to nitrocellulose membranes. The blots were blocked at room temperature (RT) for 1 h with 5% non-fat dry milk in Tris-buffered saline (TBS), followed by incubation at 4°C overnight with rabbit anti-Nrf2 (Santa Cruz, Dallas, TX, United States), anti-α-ENaC antibody (StressMarq Bioscience, Canada), and mouse anti-β-actin antibodies (Sigma-Aldrich, St. Louis, MO, United States) at a dilution of 1:1000. After being washed with TBS, blots were incubated with secondary antibodies (goat anti-rabbit IgG and rabbit anti-mouse IgG, Santa Cruz) at RT for 1 h. The immunoreactive bands were visualized using chemiluminescent reagent (Thermo Fisher Scientific) and exposed to X-ray film. Then, films were scanned and quantified using Image-Pro Plus 6.0 software.

### Quantitative Real-Time PCR (qRT-PCR)

Total RNA was isolated using TRIzol (Invitrogen), and first-strand cDNAs were synthesized from 4 μg of total RNAs in a 20-μl reaction using Superscript (Invitrogen). The first-strand cDNAs served as a template for quantitative PCR (qPCR) performed in an Applied Biosystems 7900 Real Time PCR System using SYBR green PCR reagent. Oligonucleotides were designed using Primer3 software^1^ and the sequences are shown in Table 1. Cycling conditions are 95°C for 10 min, followed by 40 repeats of 95°C for 15 s and 60°C for 1 min. The relative gene expression level was calculated through the Delta-delta Ct method and GAPDH was used as the internal control.

**TABLE 1:** The sequences of primers used in real-time PCR.

Name	Forward primer (5′–3′)	Reverse primer (5′–3′)
*GAPDH*	GTCTTCACTACCATGGAGAAGG	TCATGGATGACCTTGGCCAG
*Nrf2*	CGAGATATACGCAGGAGAGG	GCTCGACAATGTTCTCCAGCTT
	TAAGA	
*AKR7a5*	ATCAGGAGGGCAAGTTTGTG	CCAAAGGGTTGTAGGCGTAG
*AKR1a4*	GCTTAGATGGCAGGTTCAGC	AGCATCTCTGGGAACCCTCT
*HO-1*	CCTCACTGGCAGGAAATCATC	CCTCGTGGAGACGCTTTACATA
*GSTa4*	CCCCTGTACTGTCCGACTTC	GGAATGTTGCTGATTCTTGTCTT

### Statistical Analysis

All results are presented as means ± SE. The statistical analysis was performed using ANOVA followed by Bonferroni’s test or unpaired Student’s *t*-test with SPSS 13 statistical software. *p* < 0.05 was considered significant.

## Results

### Effects of Acute Salt Loading on the Protein Expression of Nrf2 in the Kidney

**FIGURE 1:**
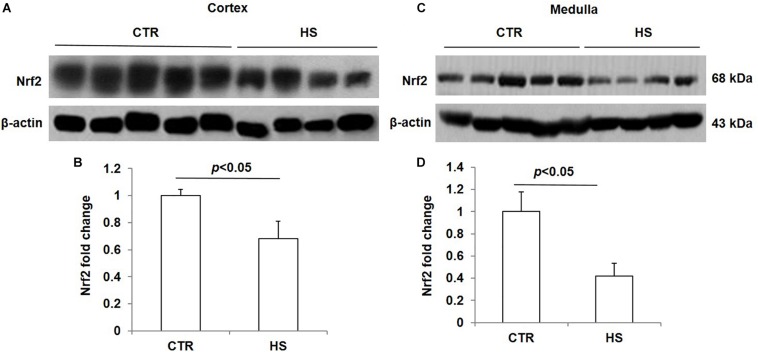
Nrf2 protein expression in kidneys after acute high salt loading. **(A)** Western blotting analysis of Nrf2 and β-actin in renal cortex. **(B)** Densitometric analysis of Nrf2 normalized by β-actin in cortex tissue. **(C)** Western blotting analysis of Nrf2 and β-actin in renal medulla. **(D)** Densitometric analysis of Nrf2 normalized by β-actin in medulla tissue. The presented data are means ± SE. Control (CTR): *n* = 5; high salt loading (HS): *n* = 4.

**FIGURE 2:**
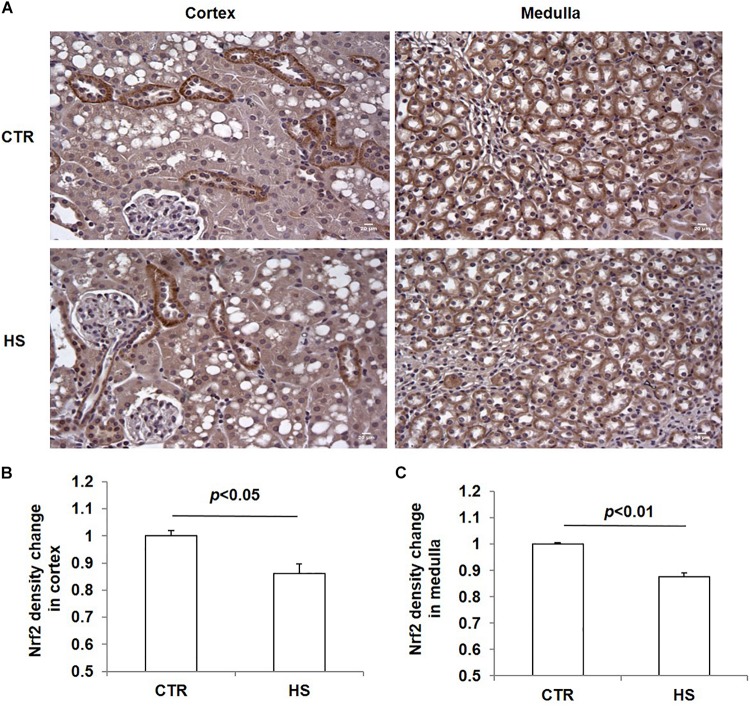
Immunohistochemistry of Nrf2 in kidneys after acute high salt loading. **(A)** Immunohistochemistry of Nrf2 in renal cortex and medulla. Magnification: 400×. Scale bar: 20 μm. **(B)** Densitometric analysis of Nrf2 in renal cortex. **(C)** Densitometric analysis of Nrf2 in renal medulla. The presented data are means ± SE. The images shown are representative of four to five animals per group.

To study the role of acute salt loading in the regulation of renal Nrf2, we examined Nrf2 protein levels in cortex and medulla tissues after 12 h of salt loading. The data showed that Nrf2 in cortex and medulla were both significantly downregulated after acute salt loading (Figures 1A–D). Immunostaining results also confirmed the significant downregulation of Nrf2 in the kidneys in response to acute high salt loading (Figures 2A–C). All these results demonstrated an inhibitory effect of acute salt loading on renal Nrf2 protein expression.

### Nrf2 Localization in the Kidney

**FIGURE 3:**
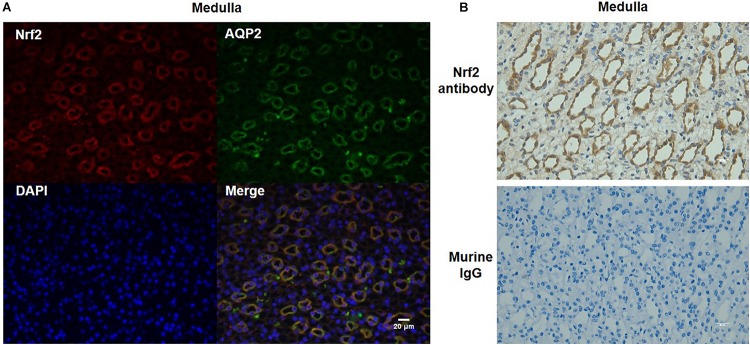
Co-localization of Nrf2 and AQP2 in normal kidneys. **(A)** Immunofluorescent co-labeling with anti-Nrf2 and anti-AQP2 antibody in normal kidneys. Nrf2 is shown as red color. AQP2 is shown as green color. DAPI staining is shown as blue color. Magnification: 400×. Scale bar: 20 μm. **(B)** Analysis of Nrf2 antibody specificity for immunostaining by replacing Nrf2 antibody with murine IgG. The images shown are representative of five normal animals.

To further define the localization of Nrf2 in the kidney, we performed immunofluorescence co-labeling of Nrf2 and AQP2 in normal kidneys. The data showed that Nrf2 was predominantly expressed in the collecting duct principal cells, overlapping with AQP2 (Figure 3A). The specificity of Nrf2 antibody for immunostaining was examined by adding murine IgG to replace Nrf2 antibody (Figure 3B). These findings suggested the Nrf2 may play a role in the collecting duct in maintaining the normal physiology of collecting ducts and renal medulla.

### Effects of Acute High Salt Loading on ROS Levels and the Protein Expression of α-ENaC in the Kidney

**FIGURE 4:**
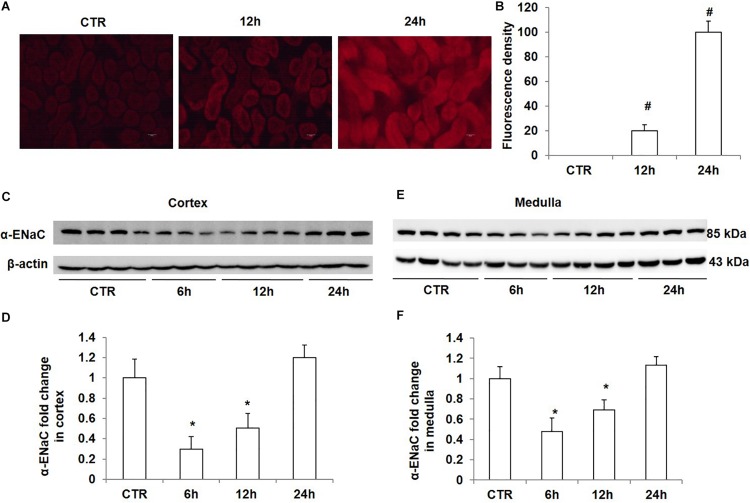
Time course analysis of ROS generation and α-ENaC protein expression in kidneys after acute high salt loading. **(A)** ROS levels were examined with DHE in the kidney after salt loading for 12 and 24 h. **(B)** Quantification of DHE fluorescence intensity in the kidney after salt loading for 12 and 24 h. **(C)** Western blotting analysis of α-ENaC and β-actin in renal cortex after salt loading for 6, 12, and 24 h. **(D)** Densitometric analysis of α-ENaC normalized by β-actin in cortex tissue. **(E)** Western blotting analysis of α-ENaC and β-actin in renal medulla after salt loading for 6, 12, and 24 h. **(F)** Densitometric analysis of α-ENaC normalized by β-actin in medulla tissue. The presented data are means ± SE. *n* = 3–4 per group. ^∗^*p* < 0.05 vs. CTR (control); ^#^*p* < 0.01 vs. CTR (control).

To study the effect of high salt loading on oxidative stress in the kidney, we examined the levels of ROS using DHE staining. As expected, ROS levels in the kidney were significantly increased after 12 h of salt loading, which were further strengthened after salt loading for 24 h (Figures 4A,B). At the same time, we measured the protein levels of α-ENaC in the kidney at different time points of salt loading (6, 12, and 24 h). The data showed that α-ENaC in both cortex and medulla were significantly decreased after 6 h or 12 h of salt loading, which restored to the control level after 24 h of salt loading (Figures 4C–F). These results demonstrated that oxidative stress was increased in the kidney after salt loading along with the downregulation of Nrf2. The downregulation of α-ENaC might be an adaptive response to salt loading. However, it remains unknown whether α-ENaC downregulation is associated with the increased ROS or decreased Nrf2 expression, which deserves further investigation.

### Effect of NaCl Treatment on Nrf2 Expression in mpkCCD Cells

**FIGURE 5:**
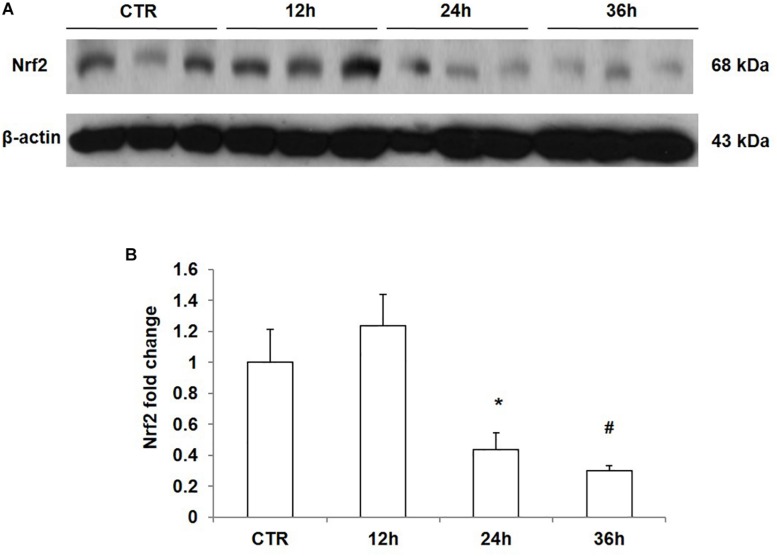
Time course analysis of Nrf2 protein expression in mpkCCD cells after sodium chloride (NaCl) treatment. **(A)** Western blotting analysis of Nrf2 and β-actin in mpkCCD cells after NaCl treatment for 12, 24, and 36 h. **(B)** Densitometric analysis of Nrf2 normalized by β-actin in mpkCCD cells after NaCl treatment for 12, 24, and 36 h. The presented data are means ± SE. *n* = 3 per group. ^∗^*p* < 0.05 vs. CTR (control); ^#^*p* < 0.01 vs. CTR (control).

To confirm the direct inhibitory effect of NaCl on Nrf2 expression, we treated mpkCCD cells with medium at 550 mOsm/kgH_2_O prepared by adding NaCl for different periods of time. The results showed that NaCl treatment for 12 h did not affect the protein expression of Nrf2; however, after treatment with NaCl for 24 h or 36 h, Nrf2 protein level was significantly downregulated in mpkCCD cells (Figures 5A,B). Taken together, NaCl directly downregulated Nrf2 expression in kidney collecting duct cells.

### Effect of NaCl, Na-Glu, Choline-Cl, and Mannitol on the Downregulation of Nrf2 in mpkCCD Cells

**FIGURE 6:**
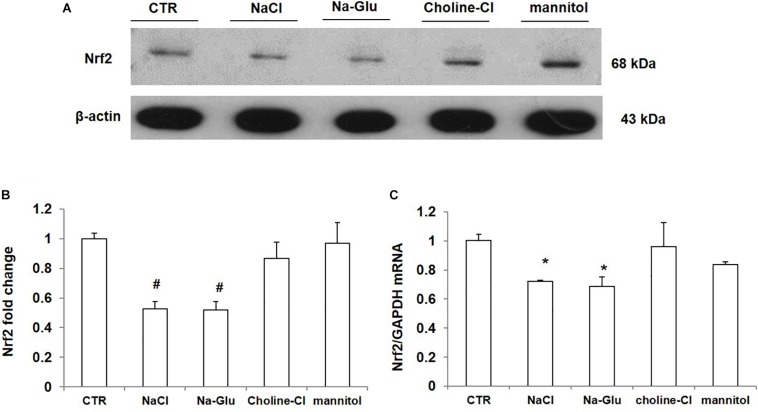
The protein and mRNA expression of Nrf2 in mpkCCD cells after the treatments of NaCl, Na-Glu, choline-Cl, and mannitol. **(A)** Western blotting analysis of Nrf2 and β-actin. **(B)** Densitometric analysis of Nrf2 normalized by β-actin. **(C)** qRT-PCR analysis of Nrf2in mpkCCD cells. The presented data are means ± SE. *n* = 3 per group. ^∗^*p* < 0.05 vs. CTR (control); ^#^*p* < 0.01 vs. CTR (control).

As shown above, NaCl treatment reduced Nrf2 expression in mpkCCD cells. We measured the protein and mRNA levels of Nrf2 after 36 h treatment with NaCl, Na-Glu, Choline-Cl, or mannitol in mpkCCD cells, aiming at defining the role of Na^+^, Cl^–^ and osmolality in mediating the downregulation of Nrf2. NaCl treatment for 36 h strikingly downregulated the protein level of Nrf2, and Na-Glu had a similar effect on Nrf2 expression as NaCl (Figures 6A,B). However, neither choline-Cl nor mannitol reduced Nrf2 protein level (Figures 6A,B). Nrf2 mRNA level was also reduced by NaCl and Na-Glu but not choline-Cl and mannitol (Figure 6C). These data suggested that the salt-induced downregulation of Nrf2 was regulated by Na^+^ rather than Cl^–^ or high osmolality.

### Effect of NaCl Treatment on the mRNA Expression of Nrf2-Targeted Genes

**FIGURE 7:**
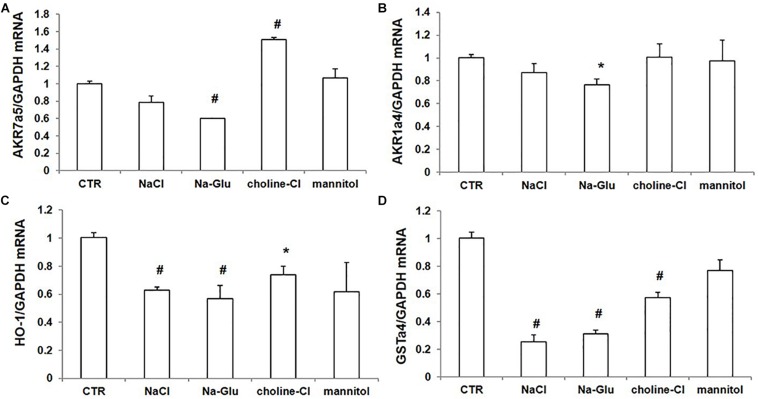
mRNA expression of Nrf2 target genes in mpkCCD cells after treatments of NaCl, Na-Glu, choline-Cl, and mannitol. **(A)** qRT-PCR analysis of AKR7a5. **(B)** qRT-PCR analysis of AKR1a4. **(C)** qRT-PCR analysis of HO-1. **(D)** qRT-PCR analysis of GSTa4. The presented data are means ± SE. *n* = 3 per group. ^∗^*p* < 0.05 vs. CTR (control); ^#^*p* < 0.01 vs. CTR (control).

We further examined the mRNA levels of some Nrf2-regulated genes via qRT-PCR. AKR7a5 mRNA level had a reduction after NaCl treatment and a significant downregulation in response to Na-Glu treatment (Figure 7A). Mannitol did not affect AKR7a5 mRNA expression, while choline-Cl significantly enhanced AKR7a5 mRNA level (Figure 7A), suggesting that choline or chloride may have a role in regulating AKR7a5 independent of Nrf2. Like AKR7a5, the mRNA expression of AKR1a4 was similarly affected by NaCl and Na-Glu (Figure 7B). However, AKR1a4 was not regulated by choline-Cl or mannitol (Figure 7B). HO-1 or GSTa4 mRNA expression was significantly decreased by both NaCl and Na-Glu, less decreased by choline-Cl, and unaltered by mannitol (Figures 7C,D). These data indicated that the mRNA levels of Nrf2-targeted genes were largely downregulated by Na^+^. Some discrepancy compared to the regulation on Nrf2 could be that these so-called Nrf2 target genes were also regulated by other transcription factors and insults.

### Effect of NAC, Spironolactone, and NS-398 on NaCl-Induced Downregulation of Nrf2 in mpkCCD Cells

**FIGURE 8:**
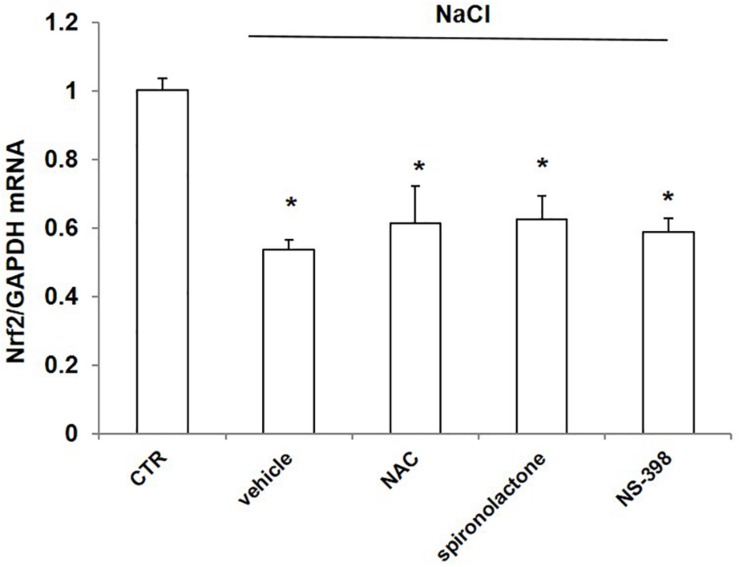
mRNA expression of Nrf2 in mpkCCD cells after treatment of NaCl in combination with NAC, spironolactone, or NS-398 treatment. All groups were treated with NaCl except CTR (control) group. NAC: N-acetylcysteine. The Nrf2 mRNA expression was determined by qRT-PCR. The presented data are means ± SE. *n* = 3 per group. ^∗^*p* < 0.05 vs. CTR (control).

To probe the possible mechanism of NaCl regulation on Nrf2, we treated mpkCCD cells with NaCl accompanied with the ROS scavenger NAC, mineralocorticoid receptor (MR) antagonist spironolactone, and COX-2 inhibitor NS-398. The Nrf2 mRNA level was remarkably decreased after NaCl treatment, which was not affected by NAC, spironolactone, or NS-398 (Figure 8). The results indicated that the downregulation of Nrf2 by NaCl treatment was independent of oxidative stress, aldosterone, and COX-2.

## Discussion

This study was designed to clarify whether high salt could affect the expression of Nrf2 and Nrf2-targeted genes, as well as determine the potential regulatory mechanism. *In vivo*, mice were treated with acute salt loading and showed that the protein levels of Nrf2 in renal cortex and medulla were markedly reduced in renal collecting ducts after an acute salt loading for 12 h. Meanwhile, the ROS levels in the kidney were increased after salt loading. The protein expression of α-ENaC in renal cortex and medulla were significantly decreased after salt loading for 6 h and 12 h, which could be a compensatory response to salt loading. Consistently, Nrf2 was also strikingly downregulated in mpkCCD cells administrated with high osmolality medium by adding NaCl. To define the roles of Na^+^, Cl^–^, and high osmolality in regulating Nrf2, we compared NaCl and Na-Glu, Choline-Cl, and mannitol treatments in mpkCCD cells. The results indicated that the downregulation of Nrf2 mRNA and protein expression could mainly depend on the effect of Na^+^ but not Cl^–^ or high osmolality. Consistently, some Nrf2 target genes showed a similar decrease in expression. To probe the potential mechanism of Na^+^ regulation on Nrf2, we treated mpkCCD cells with the antioxidant NAC, sodium channel inhibitor spironolactone, and COX-2 inhibitor NS-398. Surprisingly, none of them affected the downregulation of Nrf2 caused by NaCl, suggesting that oxidative stress, sodium transport, and COX-2-derived prostaglandins (abundant inducible mediators affecting sodium transport in kidney) may not play a role in mediating Na^+^ effect on downregulating Nrf2 in collecting duct.

Nrf2 is a well-recognized transcription factor that plays a central role in the maintenance of redox balance and protection against oxidative stress. In a completed phase 2, double blind, randomized, placebo-controlled clinical trial (NCT00811889), patients with advanced CKD and type 2 diabetes randomly assigned to bardoxolone methyl (BM), a potent Nrf2 activator, experienced significant improvement in estimated GFR (Pergola et al., 2011). Furthermore, BM ameliorated tubulointerstitial damage induced by aldosterone and salt in mice through increasing renal expression of the Nrf2 target antioxidant genes (Hisamichi et al., 2018). In the context of high NaCl, Li et al. (2017) reported that hyperosmotic medium at 400 and 450 mOsm/kgH_2_O markedly reduced the expression of Nrf2 in primary human corneal epithelial cells (HCEpiCs). Another study also showed that calcitriol protected HCEpiCs against hyperosmotic medium-induced oxidative damage by activating the translocation of Nrf2 from cytosol into nucleus and thus inducing Nrf2-regulated antioxidant enzymes (Dai et al., 2019). *In vivo* studies demonstrated that compared with normal salt diet, high-salt diet reduced Keap1 s-sulfhydration, which contributed to increased oxidative stress in the renal tissue of Dahl rats (Huang et al., 2016). In agreement with the above notion, Hosohata et al. (2019) found that a high-salt diet (8% NaCl) treatment for 4 weeks increased renal ROS. *In vitro*, high NaCl also increased ROS in human embryonic kidney 293 cells (Zhou et al., 2005). Consistently, our study confirmed that oxidative stress was increased in the kidney after acute salt loading.

At the same time, our study showed that hyperosmolarity with Na^+^ decreases mRNA and protein levels of Nrf2 in mpkCCD cells and kidneys. Besides Nrf2, we also examined the expressions of some genes regulated by Nrf2. Although these genes were downregulated by NaCl and/or Na-Glu, some of them were also regulated by Choline-Cl, indicating additional mechanisms in regulating these genes besides Na^+^ and Nrf2. Taken together, our results suggested an inhibitory effect of high Na^+^ on the expression of Nrf2 in the kidney.

Our data also demonstrated that acute salt loading for 6 and 12 h significantly decreased α-ENaC expression in kidney, which restored to the control level after salt loading for 24 h. However, Pavlov et al. (2013) found that the protein expression of β- and γ-ENaC subunits were significantly increased in the kidney cortex of Dahl salt-sensitive rats fed an 8% high-salt diet for 4 weeks, while α-ENaC expression was not changed. In contrast, in a salt-resistant rat strain (SS.13^*BN*^ rats), high-salt diet treatment for 3 weeks did not change the protein abundance of α-, β-, or γ-ENaC (Pavlov et al., 2013). Another report demonstrated that high-salt diet treatment for 18 h increased β-ENaC in Sprague–Dawley rats (Yang et al., 2008). The above findings do not agree with our data in α-ENaC expression. The discrepancy could be explained by an oral gavage approach of salt loading in the present experimental setting, which might cause stronger compensatory response in kidney to excrete salt, leading to a transient downregulation of α-ENaC. Moreover, the difference in time course (acute and chronic high salt treatment) or animal species/strains might also be involved, which needs to be studied in the future.

A high-salt diet is known to induce excessive oxidative stress in the kidney (Feng et al., 2012; Fellner et al., 2014). The local renin–angiotensin–aldosterone system (RAAS) is enhanced in Dahl salt-sensitive rats on a high-salt diet through activation of sodium channels and sodium reabsorption (Kobori et al., 2003; Gonsalez et al., 2018). High salt intake also induces abundant production of cyclooxygenase-2 (COX-2)-derived prostaglandins in the renal medulla to modulate sodium balance (Yang et al., 1998; Yang and Liu, 2017). Therefore, in the current study, we employed NAC, spironolactone, and NS-398 to define if the inhibitory effect of NaCl on Nrf2 expression was dependent on oxidative stress, aldosterone-regulated sodium transport, or COX-2-derived prostaglandins. Unexpectedly, none of them affected the downregulation of Nrf2 induced by NaCl. There might exist some unknown mechanisms for the inhibitory effect of Na^+^ on Nrf2 expression, which needs further investigation.

On the basis of our results, we conclude that high salt has an inhibitory effect on the expression of Nrf2 and Nrf2-target genes in renal collecting ducts via high concentration of Na^+^ but not Cl^–^ and hypertonicity. The findings from our current study offer new insights into the understanding of high salt effect on kidney pathology in CKD.

## Data Availability Statement

All datasets generated for this study are included in the article/Supplementary Material.

## Ethics Statement

The animal study was reviewed and approved by the Institutional Animal Care and Use Committee at Nanjing Medical University.

## Author Contributions

ZJ and XD coordinated and oversaw the study. ML, MD, and QL collected samples, performed the experimentation, and analyzed the data. ZJ, XD, and ML wrote the manuscript.

## Conflict of Interest

The authors declare that the research was conducted in the absence of any commercial or financial relationships that could be construed as a potential conflict of interest.

## Footnotes

1 http://frodo.wi.mit.edu/primer3/
